# Lateral hypothalamus orexinergic projection to the medial prefrontal cortex modulates chronic stress-induced anhedonia but not anxiety and despair

**DOI:** 10.1038/s41398-024-02860-9

**Published:** 2024-03-16

**Authors:** Danlei Liu, Xuefeng Zheng, Yuqing Hui, Yuanyuan Xu, Jinjiang Du, Zean Du, Yichen Che, Fengming Wu, Guangyin Yu, Jifeng Zhang, Xiaobing Gong, Guoqing Guo

**Affiliations:** 1 Department of Anatomy, Neuroscience Laboratory for Cognitive and Developmental Disorders, Medical College of Jinan University, Guangzhou, 510632 China; 2 Department of Gastroenterology, The First Affiliated Hospital of Jinan University, Guangzhou, 510632 China

**Keywords:** Neuroscience, Depression

## Abstract

Chronic stress-induced anxiodepression is a common health problem, however its potential neurocircuitry mechanism remains unclear. We used behavioral, patch-clamp electrophysiology, chemogenetic, and optogenetic approaches to clarify the response of the lateral hypothalamus (LH) and the medial prefrontal cortex (mPFC) to stress, confirmed the structural connections between the LH and mPFC, and investigated the role of the LH–mPFC pathway in chronic stress-induced anxiodepression symptoms. Unpredictable chronic mild stress (UCMS) caused anxiodepression-like behaviors, including anxiety, anhedonia, and despair behaviors. We discovered that the activity of the LH and mPFC was both increased after restraint stress (RS), a stressor of UCMS. Then we found that the orexinergic neurons in the LH predominantly project to the glutamatergic neurons in the mPFC, and the excitability of these neurons were increased after UCMS. In addition, overactivated LH orexinergic terminals in the mPFC induced anhedonia but not anxiety and despair behaviors in naive mice. Moreover, chemogenetically inhibited LH–mPFC orexinergic projection neurons and blocked the orexin receptors in the mPFC alleviated anhedonia but not anxiety and despair behaviors in UCMS-treated mice. Our study identified a new neurocircuit from LH orexinergic neurons to mPFC and revealed its role in regulating anhedonia in response to stress.

**Figure:**
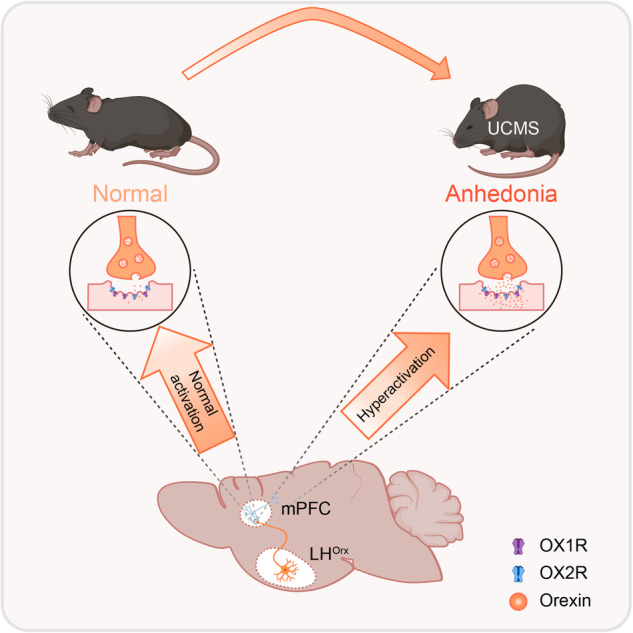
Overactivation of LH^Orx^-mPFC pathway selectively mediated chronic stress-induced anhedonia. In normal mice, the LH^Orx^-mPFC pathway exhibits relatively low activity. However, after chronic stress, the activity of orexinergic neuron in LH is overactivated, leading to an increased release of orexin into the mPFC. This heightened orexin concentration results in increased excitability of the mPFC through OX1R and OX2R, consequently triggering anhedonia.

Overactivation of LH^Orx^-mPFC pathway selectively mediated chronic stress-induced anhedonia. In normal mice, the LH^Orx^-mPFC pathway exhibits relatively low activity. However, after chronic stress, the activity of orexinergic neuron in LH is overactivated, leading to an increased release of orexin into the mPFC. This heightened orexin concentration results in increased excitability of the mPFC through OX1R and OX2R, consequently triggering anhedonia.

## Introduction

Chronic stress is known to have adverse effects on brain and behavior, and there is clear evidence that stress exposure is linked to several neuropsychiatric disorders, the most common of which are anxiety and depression [1]. Anxiodepression—that is, anxiety and depression—is a prominent determinant of the global burden of disease, often resulting in pervasive feelings of sadness, emptiness, and irritability that extend across various domains, such as academic and professional settings and family life [2]. This distress can ultimately lead to self-harming behaviors, and in severe cases, even suicide [3]. There are multiple treatments for depression and anxiety, including psychological treatments and medications. However, in up to 30–50% of patients, depression or anxiety cannot be relieved with these conventional treatments [4]. Although studies of the pathophysiological mechanisms of depression and anxiety have generated several hypotheses, the underlying etiology of these disorders remains unclear.

In recent years, there has been a growing focus on neurocircuitry disruption, which contributes to the pathogenesis of anxiodepression. New evidence suggests that orexin (Orx) plays a critical role in anxiety and depression responses to chronic stress. Orx, also commonly referred to as hypocretin, encompasses two distinct neuropeptides orexin-A (OA) and orexin-B (OB) that are predominantly synthesized and secreted by neurons located within the lateral hypothalamus (LH) [5]. Orx is involved in various biological processes, including appetite regulation, sleep, and wakefulness. Orx also participates in physiological processes such as emotion, cognition, and stress [6]. For instance, the activation of LH orexinergic neurons has been linked to anxiety responses and diminished interest in work [7]. The Orx system has the potential to enhance anhedonic behaviors by augmenting the sense of pleasure or reward associated with various stimuli, such as food intake and drug abuse [8, 9]. However, the neural circuits and molecular mechanisms underlying the regulation of anxiodepression by LH orexinergic neurons remain unclear. Consistent with their diverse physiological functions, Orx neurons project to multiple brain regions, including the medial prefrontal cortex (mPFC), anterior cingulate cortex (ACC), lateral habenula (LHb), ventral pallidum (VP), basolateral amygdala (BLA), and ventral tegmental area (VTA). The mPFC is one of the main targets of orexinergic projections and serves as a pivotal component of mood regulation [10–12]. However, there is still a lack of direct evidence regarding the role of LH^Orx^ projections to the mPFC in the progression of stress-induced anxiodepression.

In this study, we used behavioral, patch-clamp electrophysiology, chemogenetic, and optogenetic approaches to clarify the response of the LH and mPFC to stress, confirmed the structural connection between the LH and mPFC, and investigated the role of the LH^Orx^–mPFC pathway in chronic stress-induced anxiodepression symptoms, including anxiety, anhedonia, and despair behaviors.

## Materials and methods

### Animals

All animal experiments were conducted in accordance with the National Institutes of Health’s “Guide for the Care and Use of Laboratory Animals” and were approved by the Jinan University Laboratory Animal Ethics Committee (Guangzhou, China). Male C57BL/6 J mice (8 weeks old, 18–25 g) were purchased from the Beijing Vital River Laboratory Animal Technology Limited Company (Beijing, China). Prior to the start of the formal experiments, all mice were housed in home cages with a constant temperature (20–25 °C) and humidity (40–60%) located in a controlled facility under a 12 h light/dark cycle (light from 8:00 to 20:00) with food and water provided ad libitum. The mice were used for modeling or were injected with a virus after a week (at 9 weeks old) and randomly assigned to different groups. Behavioral tests were conducted in conducted in order of increasing stimulus intensity. Due to the exploratory nature of this study, no formal power analysis or sample size estimation was conducted and the sample size estimation was based on prior experience [13].

### Unpredictable chronic mild stress

**Fig. 1:**
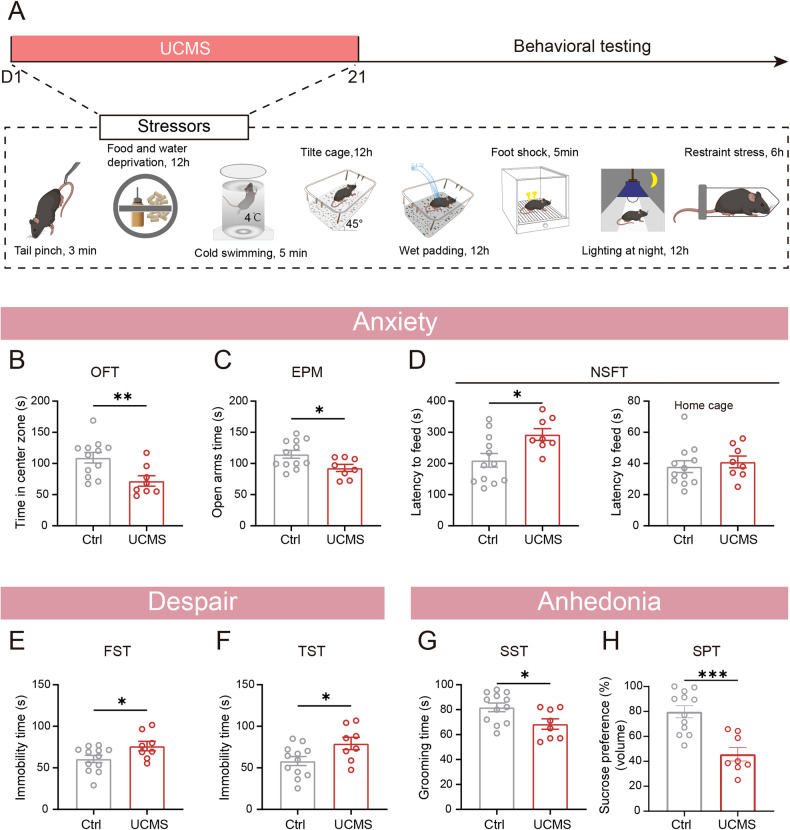
UCMS drove anxiety and depression onset. **A** The experiment protocols for UCMS, including each stressor, and the timing of behavioral tests. **B** UCMS mice showed a decreased time in the center zone in OFT. **C** The time that UCMS mice spent in the open arms was decreased in EPM. **D** The latency to feed for UCMS mice was increased in the NSFT, but not in the home cage. The immobility time of UCMS mice was increased in the FST (**E**) and TST (**F**). **G** UCMS mice exhibited a shorter grooming time in the SST than Ctrl mice. **H** UCMS mice decreased volume consumed in the SPT. *n*_(Ctrl)_ = 12, *n*_(UCMS)_ = 8. Data are shown as mean ± S.E.M., unpaired Student’s *t-*test, *P < 0.05, **P < 0.01, ***P < 0.001.

Unpredictable chronic mild stress (UCMS) was induced as previously published [14]. Mice in the UCMS group were exposed to randomized stressors twice a day for 3 weeks, including a tail pinch for 3 min, food and water deprivation for 12 h, cold swimming at 4 °C for 5 min, a 45° titling cage for 12 h, wet padding for 12 h, foot shocks for 5 min (1 mA, 5 s each time with 10 s intervals), and overnight illumination and restraint stress (RS) for 6 h (Fig. 1A). The same type of stimulation appeared discontinuously so that the mice could not predict the occurrence of the stimulation. The variability and unpredictability of the stressors are key to the success of the modeling. The control (Ctrl) group was placed in a foot shock box without an actual stimulus while the UCMS group received foot shock stress. Similarly, when the UCMS group was exposed to cold-swimming stress, the Ctrl group was placed in a cylindrical tank without water. Additionally, food and water were withheld from the Ctrl group during exposure to RS.

### Behavioral assessment

#### Open field test

The open field test (OFT) was performed as previously described [15]. Each mouse was placed in the center of a blue square box (40 × 40 × 40 cm) and allowed to explore freely for 10 min. Video recording using a camera placed directly above the chamber began immediately after the test mouse was placed in the center of the box. The time spent in the center zone, which served as an indicator of anxiety, and the total distance traveled reflecting locomotor activity, were assessed using a behavioral analysis system (TopScanLite Version 2.00).

#### Elevated plus maze test

The elevated plus maze (EPM) test was performed as previously described to measure anxiety-like behavior [16]. The maze, which was elevated 50 cm above the floor, comprised two open arms (35 × 5 cm), two closed arms (35 × 5 × 15 cm), and a central area (5 × 5 cm). Each mouse, facing one of the open arms, was placed in the central area and allowed to explore freely for 10 min. The time spent in the open arms was analyzed using a behavioral analysis system (TopScanLite Version 2.00).

#### Novelty suppressed feeding test

The novelty suppressed feeding test (NSFT) was conducted as previously described [17]. The NSFT assessed anxiety by measuring the latency of the mice to bite a food pellet in a white box after a 24 h period of food deprivation. During the test, a food-deprived mouse was introduced to a white box with a single food pellet positioned at the center, and the latency to initiate feeding was recorded over a 10 min period of time. If a mouse did not consume any food within the given 10 min timeframe, the data corresponding to that mouse were excluded from the analysis. Subsequently, the mouse was immediately placed in its home cage, with a single food pellet placed at the center of the cage. Mouse behavior was recorded for 5 min to measure latency to feed.

#### Sucrose splash test

The sucrose splash test (SST) evaluated a form of motivational behavior considered to be associated with anhedonic behavior [18–20]. Each mouse was placed in a home cage and had a 10% sucrose solution sprayed directly on its back. The total grooming duration within 5 min was recorded, including touching, scrubbing, and licking of the fur.

#### Sucrose preference test

Sucrose preference test (SPT) was performed as previously reported [21]. The mice were habituated to two bottles to drink (a 1% sucrose solution and a water, changing position every 12 h) for 24 h before the test day. Then the mice were deprived of water and food for 4 h, followed by a preference test with water and 1% sucrose for 2 h. An equipment (SA104T, SCIENCE, Nanjing, China) was used to record the duration and bouts of drinking for mice and the bottles’ weight during the test. The sucrose preference index (SPI) formula: sucrose intake/(sucrose intake + water intake) × 100%.

#### Forced swimming test

The forced swimming test (FST) was performed according to a previously reported method [13]. Each mouse was introduced into a cylindrical tank (16 cm in diameter × 28 cm in height) filled with water to a depth of 20 cm. The temperature of the water was maintained at 22–24 °C. The mice were observed for 6 min and the immobility time during the last 4 min was analyzed. In this context, immobility was defined as the absence of significant movement in the mouse’s body along the longitudinal axis, resulting in minimal water displacement.

#### Tail suspension test

The tail suspension test (TST) was conducted according to a previous method [15]. Each mouse was suspended in a box (20 × 20 × 30 cm) with tape tipping its tail (1 cm from the tip of the tail) for 6 min. Video recordings of the last 4 min were used to analyze immobility time.

#### Immunostaining and imaging

After performing the behavioral tests for 90 min, the mice were anesthetized with sodium pentobarbital (120 mg/kg) and then perfused with 30 ml of saline (0.9% NaCl, 0.9 g sodium chloride in 100 ml double distilled water, #CAS:7647-14-5, DAMAO, Tianjin, China), followed by 30 ml of 4% paraformaldehyde (PFA, #30525-89-4, Sigma-Aldrich, Missouri, USA). The mouse brain was quickly removed and placed in a tube containing 4% PFA at 4 °C, where it remained overnight. Following gradient dehydration with 15% and 30% sucrose solution (#57-50-1, Sigma-Aldrich) in a phosphate buffer (0.2 M PB) at 4 °C, the mice brains were sliced (30 μm) by a rapid sectioning cryostat (CM1900, LEICA, Weztlar, Germany) and were blocked for 1 h at room temperature with 3% bovine serum albumin (BSA, #CAS9048-46-8, GENVIEW, Florida, USA) and 1% Triton X-100 (#CAS9002-93-1, BIOFROXX, Germany) in 10 ml of phosphate buffered saline (0.1 M PBS, #P4417, Sigma-Aldrich). The brain slices were first incubated in 1% BSA and 1% Triton X-100 in PBS with a rabbit anti-c-Fos antibody (1:300, #mAB2250s, Cell Signaling Technology, Boston, USA), mouse anti-orexin-A (1:500, #sc80263, Santa Cruz), rabbit anti-glutamate (1:500, #G6642, Sigma-Aldrich), mouse anti-Orexin R-1 (1:100, #sc-166111, Santa Cruz), goat anti-Orexin Receptor 2 (1:500, #EB08124, Everest Biotech, Oxfordshire, UK) and rabbit anti-GABA (1:500, #A2052, Sigma-Aldrich) for 48 h at 4 °C. The brain slices were then incubated with a secondary antibody, goat anti-mouse Alexa Fluor 555 (1:800, #A31570, Invitrogen, California, USA), goat anti-rabbit Alexa Fluor 555 (1:800, #A21428, Invitrogen), donkey anti-goat Alexa Fluor 488 (1:800, #A11055, Invitrogen), goat anti-rabbit Alexa Fluor 488 (1:800, #A21206, Invitrogen), donkey anti-rabbit Alexa Fluor 647 (1:800, #A31573, Invitrogen) for 2 h at room temperature. Finally, the brain slices were mounted with the DAPI-Fluoromount-G aqueous mounting medium (#17985-50, Electron Microscopy Sciences, Hatfield, UK). Fluorescent images of these slices were taken using a microscope (DMI8 LED, Leica).

#### Immunohistochemistry

The section preparation method for the mouse brain has been mentioned above. First, the sections were incubated in 0.3% H_2_O_2_ at room temperature for 30 min for quenching; then, the sections were incubated in rabbit anti-c-Fos (1:300, #mAB2250s, Cell Signaling Technology) for 48 h at 4 °C. The sections were then incubated with Biotinylated Anti-Rabbit IgG (1:200, #BA1000, Vector Laboratories, Newark, USA) at room temperature for 2 h before being incubated in an avidin-biotin-complex (1:100, #PK6100, Vector Laboratories) for 2 h at room temperature. Finally, the sections were incubated in diaminobenzidine (DAB, 20 × DAB 50 μl in 1 ml substrate solution, #ZLI-9019, ZSGB-BIO, Beijing, China) for 5 min. Between each step, the sections underwent three consecutive 10 min washes with 0.1 M PBS. After drying on glass slides, the sections were dehydrated using graded ethanol, vitrified with dimethylbenzene, and covered with resin. Images were captured using a microscope slide scanner (PANNORAMIC MIDI II, 3DHISTECH Ltd., Budapest, Hungary).

#### Stereotaxic surgery, virus injection, and optic fiber and cannula implantation

The mice were anesthetized with isoflurane (5% for induction, 1–2.5% for maintenance, #R510-22, RWD, Shenzhen, China) and restricted using stereotaxic equipment (#68045, RWD). Erythromycin ointment was applied to the eyes of the mice to prevent dryness. We then made an incision on the mouse scalp to expose the skull before injecting the virus into the target brain area (LH: AP − 1.10 mm, ML − 1.05 mm, DV − 5.2 mm, mPFC: AP − 0.33 mm, ML + 2.10, DV − 2.10 mm, ACC: AP − 0.30 mm, ML + 1.25 mm, DV − 1.40 mm) using a glass pipette connected to a nanoliter injector with a controller (LEGATO 130, RWD) mounted on stereotaxic equipment. The volume of the injected virus was between 0.15 μl and 0.25 μl per side, and the speed was maintained at a rate of 0.05 μl/min. The pipette was removed 10 min after infusion. To enable optogenetic activation and in vivo calcium signal recording, an optic fiber (200 μm in diameter, 0.37 NA, Inper Tech, Hangzhou, China) with a ceramic ferrule was implanted into the mPFC or LH at the previously determined coordinates. To microinjection of drugs, a cannula (#62004, RWD) was implanted into the mPFC. The optic fiber and cannula were firmly secured to the skull using three skull screws (1 mm diameter, 3 mm length, Inper Tech) positioned around the craniotomy site, and dental cement was applied for additional stability.

#### Microinjection of drugs

Microinjection using a micropump was carried out 30 min before the behavioral tests. The Orx 1 receptor (Ox1R) antagonist SB334867 (SB, 25 mM, #1455, Tocris Bioscience, Bristol, UK) and Orx 2 receptor (Ox2R) antagonist TCS-OX2-29 (TCS, 33.3 μg/μL, #1457, Tocris Bioscience) dissolved in dimethyl sulfoxide (DMSO, #196055, MP Biomedicals, California, USA) and saline were injected to the mPFC, respectively [22].

#### Retrograde non-trans synaptic tracing

AAV2/9-hEF1α-DIO-EYFP-WPRE-pA (0.3 μl, 1 × 10^13^ vg/ml, #S0196-9, Taitool Bioscience, Shanghai, China) and AAV2/Retro-hSyn-CRE-WPRE-pA (0.3 μl, 2.42 × 10^12^ vg/ml, #PT-0136, BrainVTA, Wuhan, China) were respectively injected into the LH and mPFC to label the LH neurons projecting to the mPFC.

#### Anterograde transsynaptic tracing

We injected AAV2/9-Hypocretin-CRE-WPRE-pA (0.1 μl, 5.31 × 10^12^ vg/ml, #PT-1573, BrainVTA) and AAV-EF1α-DIO-EGFP-T2A-TK(herpes simplex virus, HSV) (0.1 μl, 3.26 × 10^12^ vg/ml, #BC-0045, Brain Case, Shenzhen, China) into the LH. Subsequently, after a 21-day interval, H129ΔTK-CAG-LSL-tdTomato (0.2 μl, 2.00 × 10^12^ IFU/ml, #BC-HSV-H361, Brain Case) was injected into the LH to label the mPFC neurons receiving projection from the orexinergic neurons in LH .

In a separate procedure, AAV2/1-hSyn-Cre-WPRE-pA (0.3 μl, 1 × 1013 vg/ml, #S0196-9, Taitool Bioscience) and AAV2/9-hSyn-DIO-mCherry-WPRE-pA (0.3 μl, 1.53 × 10^13^ vg/ml, #S1139-9, Taitool Bioscience) were respectively injected into the LH and mPFC to label the mPFC neurons receiving projection from the LH.

#### Detection of synaptic connections

The mammalian green fluorescent protein (GFP) reconstitution across synaptic partners (mGRASP) was used to analyze the synaptic connection of the LH-mPFC pathway [23]. This technique relies on functional complementation involving two distinct, non-fluorescent split-GFP fragments targeted to the pre- and post-synaptic membrane. These fragments reassemble into a fluorescent GFP configuration within the targeted location, which occurs when two neurons, each expressing one of the fragments, are in close proximity across a synaptic cleft. For this analysis, AAV2/9-CAG-pre-mGRASP-mCerulea-WPRE-SV40 polyA (0.3 μl, 2.91 × 10^12^ vg/ml, #PT-3982, BrainVTA) was injected into the LH, and AAV2/9-CAG-post-mGRASP-T2A-dTomato-WPRE-SV40 polyA (0.2 μl, 2.48 × 10^12^ vg/ml, #PT-3983, BrainVTA) was administered into the mPFC. After 21 days, mice were euthanized for the examination and observation of the target brain region. The presence of GFP indicated the existence of monosynaptic connections.

#### Fiber photometry recording

AAV2/9-Hypocretin-CRE-WPRE-pA (0.2 μl, 5.31 × 10^12^ vg/ml, #PT-1573, BrainVTA) and AAV2/9-hSyn-DIO-GCaMP6m-WPRE-pA (0.3 μl, 2.30 × 10^13^ vg/ml, # S0277-9, Taitool Bioscience) were injected into the LH using an optical fiber (200 μm diameter and 0.37 NA, Inper Tech) for in vivo calcium signal recording.

AAV2/9-hSyn-Orexin0.4-WPRE-hGH-pA (0.3 μl, 2.0 × 10^12^ vg/ml, #PT-3985, BrainVTA) was injected and an optical fiber (200 μm diameter and 0.37 NA, Inper Tech) was implanted in the mPFC to record in vivo Orx release.

One week after virus injection, the mice were randomly divided into UCMS and Ctrl groups, followed by three weeks of UCMS modeling. After modeling, a fiber photometry system (FPS-410/470, Inper Tech) equipped with 470 nm and 410 nm excitation lasers was used to record the fluorescence signals of the Ca^2+^ responses of the orexinergic neurons and the concentration of orexin in the mPFC during the behavioral tests. The 470 nm signal represented fluorescence from GcaMP6m or orexin 0.4 when measuring neuronal activity, while the 410 nm signal represented a background for movement and bleaching. Inper Plot (Inper Ltd.) and a customized MATLAB code (MathWorks) were used to analyze the fluorescence change ratios and raw data. F was the signal after bleaching and motion correction, which was obtained by subtracting the 410 nm signals from the 470 nm signals. F0 was the average baseline fluorescence signal recorded before the behavioral tests. The fluorescence change values (ΔF/F) were calculated using the formula: (F-F0)/F0, which was represented as average plots or heat maps. Each row represents a trial in the heat map.

#### Chemogenetic manipulations

AAV2/9-Hypocretin-CRE-WPRE-pA, AAV2/Retro-hSyn-DIO-hM3D(Gq)-mCherry-WPRE-pA (0.3 μl, 1.53 × 10^13^ vg/ml, #S0192-2RP, Taitool Bioscience), AAV2/Retro-hSyn-DIO-hM4D(Gi)-mCherry-WPRE-pA (0.3 μl, 1.29 × 10^13^ vg/ml, #S0193-2RP, Taitool Bioscience) and AAV2/Retro-hEF1α-DIO-mCherry-WPRE-pA (0.3 μl, 1 × 10^13^ vg/ml, #S0193-2RP, Taitool Bioscience) were used for in vivo chemogenetic activation and inhibition. Three weeks after viral injection, 45 min before the behavioral tests, the mice were intraperitoneally injected with clozapine-N-oxide (CNO, 3.3 mg/kg in saline, #C0832, Sigma-Aldrich) activating hM3Dq-expressing neurons and inhibiting hM4Di-expressing neurons.

#### Optogenetic manipulations

A 465 nm blue light laser (5 mW, 5 ms, 30 Hz, B2-465, Inper Tech) was used to activate the LH orexinergic neuron terminals projecting to the mPFC. AAV2/9-Hypocretin-CRE-WPRE-pA (0.3 μl, 2.42 × 10^12^ vg/ml, #PT-1573, BrainVTA) and AAV2/9-hEF1α-DIO-hChR2(H134R)-EYFP-WPRE-pA (0.3 μl, 1 × 10^13^ vg/ml, #S0199-9-H20, Taitool Bioscience) or AAV2/9-hEF1α-DIO-EYFP-WPRE-pA (0.3 μl, 1 × 10^13^ vg/ml, #S0196-9, Taitool Bioscience) were injected into the LH and an optic fiber was implanted in the mPFC.

#### Slice preparation

Mice were anesthetized and perfused with 20 ml ice-cold high glucose artificial cerebrospinal fluid (ACSF, composition in mM:26 NaHCO_3_, 2.6 KCl, 1.23 NaH_2_PO_4_, 10 MgCl_2_, 10 glucose, 212.7 sucrose and 0.5 CaCl_2_, oxygenated with 95% O_2_ and 5% CO_2_). Following this, 300 μm coronal brain slices were prepared using a fully automatic vibrating slicer (VT1200S Leica) in ice-cold ACSF (containing in mM: 26 NaHCO_3_, 3 KCl, 1.25 NaH_2_PO_4_, 10 glucose, 124 NaCl, 1 MgCl_2_, and 2 CaCl_2_). Slices were recovered for 30 min at 34 °C in ACSF saturated with 95% O_2_ and 5% CO_2_ before recording. Sections were stored at room temperature until further analysis.

#### In vitro electrophysiology

Whole-cell patch-clamp recordings of the LH were performed according to a previously reported method [13]. Slices were transferred to a recording chamber perfused with O_2_-saturated ACSF at a rate of 2 ml/min (25 °C). LH fluorescent neurons were visualized using an upright microscope (Nikon Eclipse FN1, Nikon, Tokyo, Japan) equipped with an infrared-sensitive camera and a × 40 water immersion lens. The recording electrodes (4–6 MΩ) were filled with an internal solution comprising the following (in mM): 10 KCl, 130 K-gluconate, 130 potassium gluconate, 10 HEPES, 0.5 GTP, 4 ATP, 0.2 EGTA, and 10 Na-phosphocreatine (pH 7.2, 300 mOsm). An integrated patch-clamp amplifier (Sutter Instrument, Novato, CA, USA) was used to record the electrophysiological signals, which were digitized at 10 kHz and Bessel filtered at 2 kHz.

To confirm the validity of ChR2 activation, 465 nm blue light (5 ms, from 5 to 30 Hz) was used to elicit action potential recorded in current-clamp mode through an optical fiber coupled to a LED light source above the recorded ChR2-expressed cell.

To determine the efficacy of CNO on the hM3Dq and hM4Di virus, we recorded hM3Dq-expressing and hM4Di-expressing cells in the current-clamp mode. First, a constant current was injected into the cells to record the baseline using ACSF. Following this, 1 μM CNO was added to the ACSF. As a result, the hM3Dq-expressing neurons became depolarized and the excitability of the cells was increased compared to the baseline. On the contrary, the hM4Di-expressing neurons became hyperpolarized and the excitability of the cells was reduced compared to the baseline. This phenomenon was blocked by elution with normal ACSF.

To assess the activity of LH^Orx←mPFC^ neurons and the mPFC neurons, we employed the spike number at a consistent current intensity and the minimum current required to elicit an action potential (rheobase current). This approach allowed us to quantify the excitability of the orexinergic neurons in the LH projecting to mPFC and the mPFC. To record LH^Orx←mPFC^ neurons, AAV2/Retro-Hypocretin-CRE-WPRE-hGH-pA (0.2 μl, 5.31 × 10^12^ vg/ml, #PT-1573, BrainVTA) was injected into the mPFC and AAV2/9-hSyn-DIO-mCherry-WPRE-pA (0.3 μl, 1.53 × 10^13^ vg/ml, #S1139-9, Taitool Bioscience) was injected into the LH. The rheobase, characterized as the minimal current required to initiate a single action potential, was determined through suprathreshold current injections in the current-clamp mode. Incremental depolarizing currents ranging from 0 to 300 pA (500 ms duration) were applied in 25 pA increments. Measurement of the rheobase current was conducted in the current-clamp mode by introducing depolarizing pulse steps (2 pA increments, 500 ms duration) until the induction of action potentials.

#### Quantification statistical analyses

All experimental data were analyzed using the GraphPad Prism software 9.3 (GraphPad Software, San Diego, CA, USA). Animals or data were excluded from the analysis if there were inaccuracies in the viral infection, optic fiber and cannula implantation. The researchers conducted blinded measurements on different experimental groups. Normal distribution was assessed using the Shapiro–Wilk *W* test, and if applicable, Student’s *t*-tests and one-way ANOVA were employed for data analysis. If not applicable, a logarithmic transformation was applied to the data to achieve normality before conducting statistical analyses. Data was presented as mean ± S.E.M. and statistical significance was set as *P* < 0.05.

## Results

### UCMS induced anxiety, anhedonia and behavior despair in mice

First, we examined whether UCMS could lead to anxiodepressive behaviors in mice (Fig. 1A). After randomly exposing the mice to eight different stressors for 3 weeks, the results of the OFT showed that, compared with the mice in the Ctrl group, mice in the UCMS group spent significantly less time in the center of the box (Fig. 1B), but no significant differences were found in the total distance traveled (Supplementary Fig. 1A). In the EPM test, the time spent in the open arms was significantly shorter for the UCMS-treated mice than it was for the Ctrl group (Fig. 1C). In the NSFT, the UCMS group exhibited increased latency to bite food pellets in a novel environment compared to the Ctrl group, and no significant differences were noted in the home cage setting (Fig. 1D). These results indicate that UCMS induces anxiety-like behavior in mice. Next, the effects of UCMS on behavioral despair were tested. The UCMS-treated mice displayed increased immobility time in both the FST and TST (Fig. 1E, F). In addition, mice in the UCMS group exhibited a significant decrease in grooming time during the SST (Fig. 1G). Additionally, UCMS led to a decrease in sucrose preference indicators, including volume consumed, drinking duration but not affected drinking bouts in the SPT (Fig. 1H and Supplementary Fig. 1B), suggesting the presence of anhedonia after UCMS exposure. In summary, we confirmed that UCMS induced anxiety, anhedonia, and despair in mice.

### The neuronal activity of LH^Orx^ was closely related to UCMS-induced anxiodepression

**Fig. 2:**
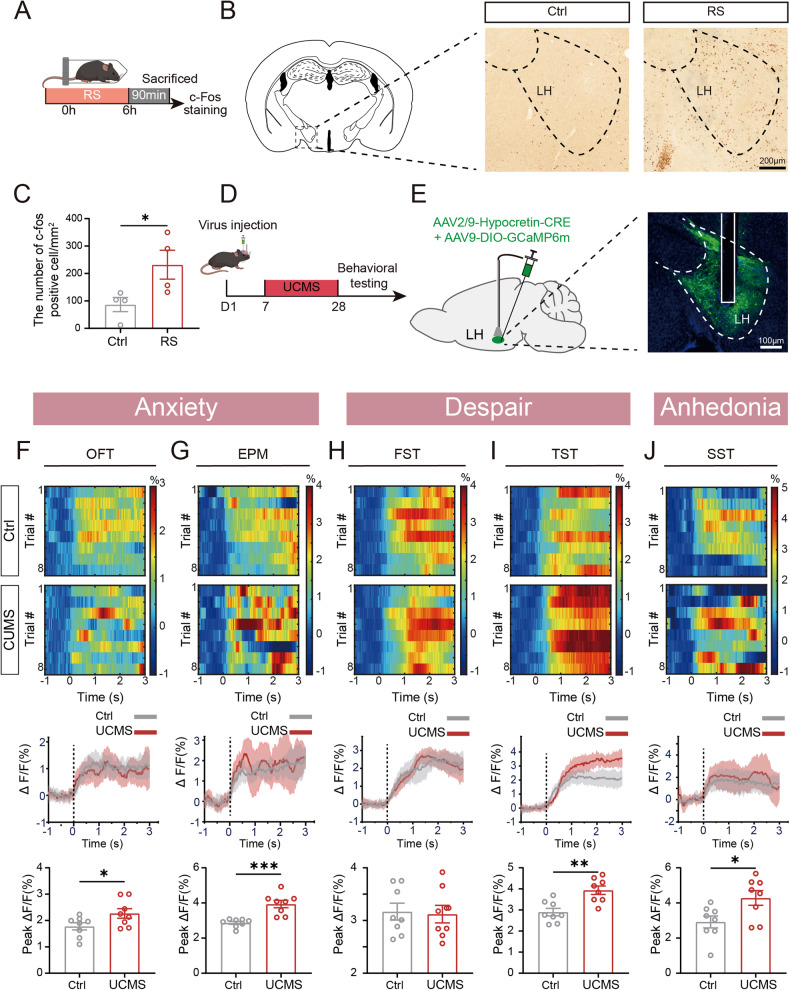
The neuronal activity of LH^Orx^ was closely related to UCMS-induced anxiodepression. **A** The experimental protocol for the activity of the LH after RS. **B** Representative c-Fos positive cells in the LH of Ctrl and RS mice. **C** The density of c-Fos positive cells in the LH region (*n* = 4). **D** The experimental protocol for in vivo fiber photometry. **E** Left: schematic of the viral injection; Right: representative virus expression image of the LH injection site. **F** Heatmaps (top) and averaged responses (middle) showing Ca^2+^ responses evoked by mice entering the center zone in the OFT; bottom: the activity of orexinergic neurons significantly increased the amplitude of Ca^2+^ signals associated with exposure to the center zone in the OFT. **G** Heatmaps (top) and averaged responses (middle) showing Ca^2+^ responses evoked by mice entering the open arms in the EPM; bottom: activity of orexinergic neurons significantly increased the amplitude of Ca^2+^ signals associated with exposure to the open arms in the EPM. **H** Heatmaps (top) and averaged responses (middle) showing Ca^2+^ responses evoked by mice struggling in the FST; bottom: the amplitude of Ca^2+^ signals in orexinergic neurons associated with struggling in the FST was no significiant difference between two groups. **I** Heatmaps (top) and averaged responses (middle) showing Ca^2+^ responses evoked by mice struggling in the TST; bottom: activity of orexinergic neurons significantly increased the amplitude of Ca^2+^ signals associated with exposure to struggling in the TST. **J** Heatmaps (top) and averaged responses (middle) showing Ca^2+^ responses evoked by mice grooming in the SST; bottom: activity of orexinergic neurons significantly increased the amplitude of Ca^2+^ signals associated with exposure to grooming in the SST after UCMS. **F**–**J** 8 trails from 3 mice. Data are shown as mean ± S.E.M., unpaired *t* test, *P < 0.05, **P < 0.01, ***P < 0.001.

Prior studies have provided evidence supporting the crucial role of LH orexinergic neurons in the regulation of anxiodepression [24]. RS is one of the stressors in UCMS, often employed as a singular stressor to induce anxiety- and depression-like behaviors [15, 25]. To clarify the role of LH orexinergic neurons in anxiodepression-like behaviors induced by stress, c-Fos immunofluorescence staining was used to detect the response of the LH to RS (Fig. 2A). The results revealed a notable increase in the number of c-Fos-immunopositive cells in the LH following exposure to RS, suggesting that the LH neurons were activated by stress (Fig. 2B, C).

To further investigate the correlation between the hyperactivation of LH orexinergic neurons and anxiodepression, we employed an experimental approach involving the injection of AAV2/9-DIO-GCaMP6m and AAV2/9-Hypocretin-CRE into the LH, as well as the insertion of an optic fiber. Subsequently, we used in vivo fiber photometry to record calcium signaling in orexinergic neurons at the soma level (Fig. 2D, E). After UCMS, orexinergic neuronal Ca^2+^ levels were elevated during mice entering the central zone of the OFT and the open arms of the EPM test (Fig. 2F, G). Compared with the Ctrl group, the orexinergic neuronal Ca^2+^ level in the UCMS group could also be elevated when transitioning from immobility to struggle in the TST (Fig. 2I), but not in the FST (Fig. 2H). Meanwhile, the calcium signals of orexinergic neurons were elevated in response to UCMS during grooming in the SST (Fig. 2J). These results indicate that LH orexinergic neurons exhibit increased activity after the presentation of anxiodepression.

### The activity of the mPFC neurons receiving projections from orexinergic neurons was activated by stress

**Fig. 3:**
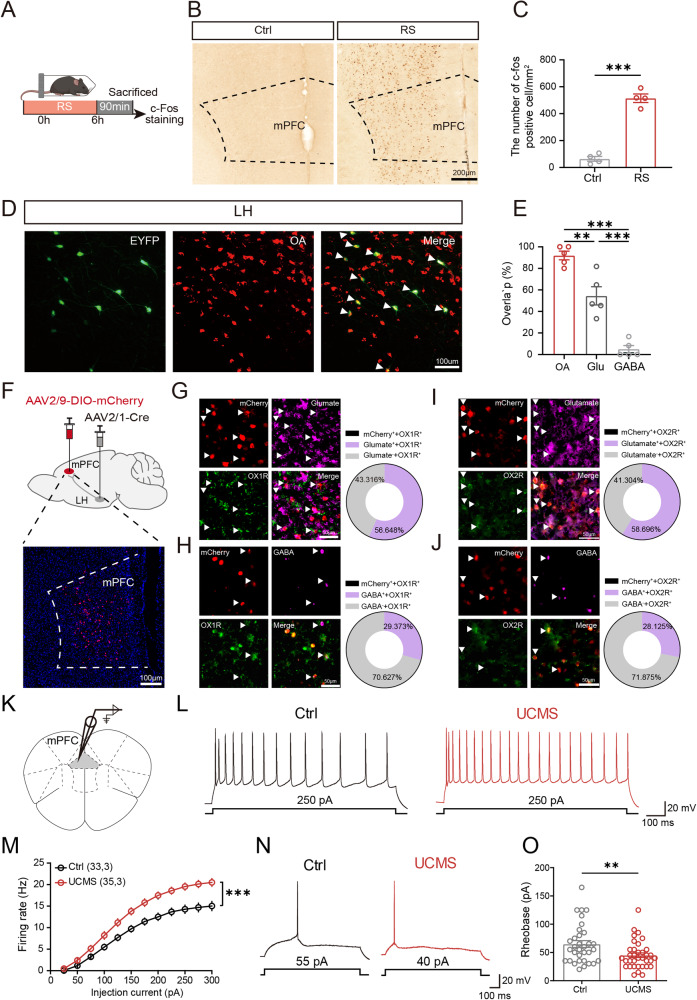
The activity of the mPFC neurons receiving projections from orexinergic neurons was activated by stress. **A** The experimental protocol for the activity of mPFC after RS. **B** Representative c-Fos positive cells in the mPFC for mice in the Ctrl and RS groups. **C** The density of c-Fos positive cells in the mPFC region (*n* = 4). **D** Representative photomicrographs of EYFP expression overlapped with neurons expressing orexin-A. **E** Percentage of EYFP neurons expressing orexin-A, Glu, and GABA (*n* = 4). **F** Top: schematic viral injection plan; Bottom: representative virus expression image in the mPFC. **G** Left: Representative photomicrographs of mCherry expression overlapped with neurons expressing OX1R and Glu. Right: Percentages of Glu neurons receiving the orexinergic neuron projection in mPFC. **H** Left: Representative photomicrographs of mCherry expression overlapped with neurons expressing OX1R and GABA. Right: Percentages of GABA neurons receiving the orexinergic neuron projection in mPFC. **I** Left: Representative photomicrographs of mCherry expression overlapped with neurons expressing OX2R and Glu. Right: Percentages of Glu neurons receiving the orexinergic neuron projection in mPFC. **J** Left: Representative photomicrographs of mCherry expression overlapped with neurons expressing OX2R and GABA. Right: Percentages of GABA neurons receiving the orexinergic neuron projection in mPFC. **K** Scheme of experiments. Whole-cell patch-clamp recording on mPFC^Glu^ neurons. **L** Presentative trace of action potential produced by 250 pA current injection. **M** A line chart showed the number of action potentials evoked by inward currents in Ctrl and UCMS (Two-way analysis of variance, post hoc least significant difference test, F_1,66_ = 17.47, *P* < 0.0001). **N** Presentative trace of single action potential induced by the minimal injection current (rheobase). **O** Scatter plot showed all individual data of the rheobase current (*n*_Ctrl_ = 33, *n*_UCMS_ = 35, from 3 mice, unpaired *t* test, *P* = 0.0092). Data are shown as mean ± S.E.M., unpaired *t* test, *P < 0.05, **P < 0.01, ***P < 0.001.

LH orexinergic neurons project to multiple brain regions to regulate various functions [26]. Of particular significance is the fact that the mPFC, one of downstream brain region of the LH is a core area of anxiodepression regulation [27]. To investigate the involvement of the mPFC in stress-induced anxiodepression, we initially examined whether stress regulates the excitability of the mPFC. Mice were subjected to a 6-h RS, and c-fos staining was employed to observe the response of the mPFC (Fig. 3A). The results showed the number of c-Fos-positive cells in the mPFC increased after RS, indicating that the mPFC may be involved in the regulation of stress (Fig. 3B, C). Hence, it was inferred that both LH and mPFC were involved in the stress-induced anxiodepression. Therefore, we further elucidated the synaptic connection between the LH and mPFC.

To clarify whether there is a neural connection between these two nuclei, we injected AAV2/Retro-CRE into the mPFC and AAV2/9-DIO-EYFP into the LH (Supplementary Fig. 2A). A large number of neuron somas expressing EYFP were found in the LH, indicating that there were neurons in the LH that projected to the mPFC. To further verify the type of neurons in the LH projecting to the mPFC, orexin-A, glutamate (Glu), or GABA were stained in the LH (Fig. 3D and Supplementary Fig. 2B). We found that the colocalization ratio of EYFP-expressing LH neurons was 91.97 ± 3.98% with orexin-A, 54.47 ± 8.47% with Glu, and 5.00 ± 3.33% with GABA, respectively (Fig. 3E). Therefore, the neurons projecting to the mPFC from the LH were predominantly orexinergic neurons.

To verify structural synaptic connections of the LH-mPFC pathway, we used the mGRASP system, which enables mapping mammalian synaptic connectivity with light microscopy. This technique split the GFP into a non-fluorescent gene fragment targeted specifically to the pre- and post-synaptic membrane. When each of the two neurons expresses one of the fragments, the GFP can be reconstituted and observed at their synaptic cleft. AAV2/9-CAG-pre-mGRASP-mCerulea and AAV2/9-CAG-post-mGRASP-dTomato were respectively injected into the LH and the mPFC (Supplementary Fig. 2C, D). Consequently, we detected reconstituted GFP signals between the structures of presynaptic LH neuronal axons and a postsynaptic mPFC neuron, demonstrating LH-mPFC synaptic connections (Supplementary Fig. 2D). Then, to explore the projection of orexinergic neurons in the LH, we injected AAV2/9-Hypocretin-CRE and AAV-EF1α-DIO-EGFP-T2A-TK (HSV) into the LH, which was necessary for HSV crossing synapses in orexinergic neurons in the LH. Then H129ΔTK-CAG-LSL-tdTomato was injected into the LH after 21 days, ensuring that HSV only crosses synapses within orexinergic neurons, reaching the next level of neurons and expressing tdTomato (Supplementary Fig. 2E). We observed EGFP^+^ neurons and HSV^+^ neurons in the LH, indicating that HSV infected successfully (Supplementary Fig. 2F). Meanwhile HSV^+^ neurons were observed in the mPFC and ACC (Supplementary Fig. 2G, H), indicating that orexinergic neurons could project to the mPFC and ACC.

The mPFC is predominantly composed of pyramidal neurons, with a small population of GABAergic neurons. In order to identify the cell type of the mPFC that received the projection of LH^Orx^ neurons, we injected AAV2/1-Cre into the LH and AAV2/9-DIO-mCherry into the mPFC, finding a large number of mCherry^+^ neurons in the mPFC (Fig. 3F). We employed immunofluorescence with mCherry^+^ neurons labeled separately with OX1R or OX2R, along with Glu or GABA. Initially, we labeled cells co-infected with OX1R or OX2R and the mCherry^+^ neurons. Subsequently, we assessed the proportion of these cells co-labeled with Glu or GABA, revealing that the majority of co-labeled cells were glutamatergic neurons, with a smaller fraction being GABAergic neurons (Fig. 3G–J). Therefore, the orexinergic neurons predominantly project to the glutamatergic neurons in the mPFC.

So, were these glutamatergic neurons the cause of stress-induced anxiodepressive behaviors? We further used electrophysiological methods to record the excitability of the mPFC^Glu^ neurons after UCMS (Fig. 3K). The results indicated that the number of evoked action potentials of mPFC^Glu^ neurons in response to depolarizing current significantly increased after UCMS (Fig. 3L, M). The rheobase of evoked single action potential of mPFC^Glu^ neurons was obviously lower after UCMS (Fig. 3N, O). These results indicated that the excitement of the mPFC^Glu^ neurons was increased after UCMS.

### The excitability of the LH^Orx^-mPFC pathway was increased after UCMS

**Fig. 4:**
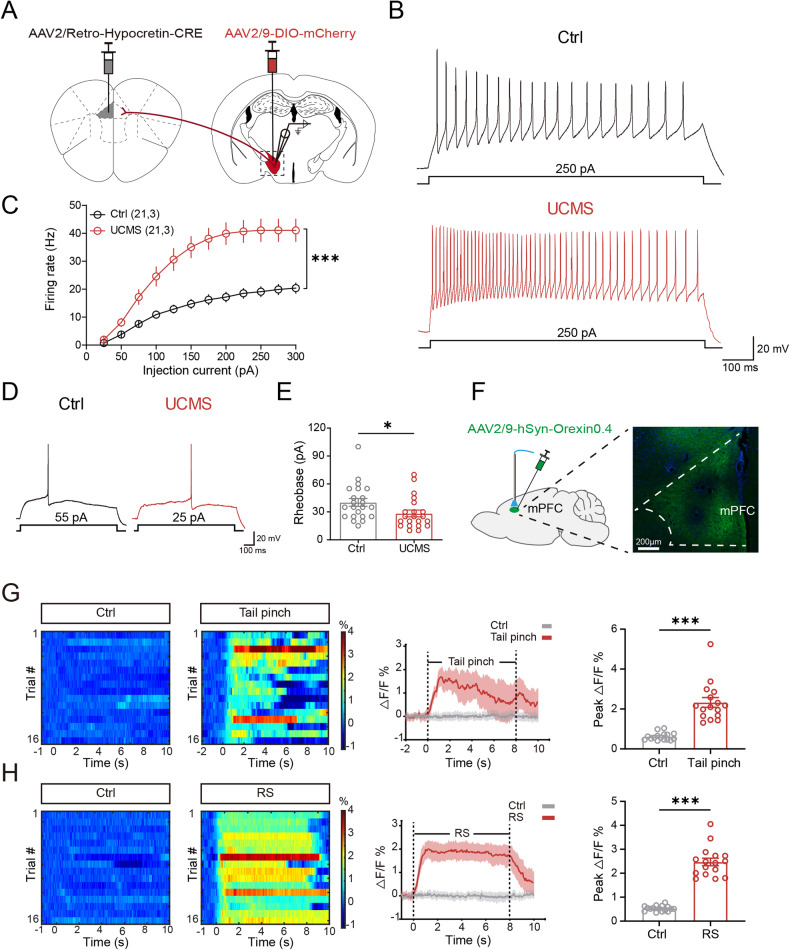
The excitability of LH^Orx^-mPFC pathway was increased after UCMS. **A** Scheme of experiments. Whole-cell patch-clamp recording on mCherry-expressing neurons in LH. **B** presentative trace of action potential produced by 250 pA current injection. **C** A line chart showed the number of action potentials evoked by inward currents in Ctrl and UCMS (Two-way analysis of variance, post hoc least significant difference test, F_1,41_ = 26.44, *P* < 0.0001). **D** Presentative trace of single action potential induced by the minimal injection current (rheobase). **E** Scatter plot showed all individual data of the rheobase current (*n*_Ctrl_ = 21, *n*_UCMS_ = 21, from 3 mice, unpaired *t* test, *P* = 0.0403). **F** Left: schematic of viral injection; Right: representative virus expression image in the mPFC. **G** Heatmaps (left) and averaged responses (middle) showing the concentration of orexin signaling evoked by a tail pinch; Right: the concentration of orexin signaling significantly increased during the tail pinch. **H** Heatmaps (left) and averaged responses (middle) showing the concentration of orexin signaling evoked by RS; Right: the concentration of orexin signaling significantly increased the amplitude of signals associated with RS. **G**–**H** 16 trails from 3 mice. Data are shown as mean ± S.E.M., *P < 0.05, ***P < 0.001.

In accordance with the results mentioned above, we hypothesized that the LH and mPFC cooperated to regulate stress-induced behavioral abnormalities. To identify the role of LH^Orx^-mPFC pathway after UCMS, firstly we used virus trace system to mark the LH orexinergic neurons projecting to the mPFC and detected the excitability of neurons by electrophysiological methods (Fig. 4A). According to the results, compared with the Ctrl group, the number of elicited action potential of LH^Orx←mPFC^ neurons in response to depolarizing current significantly increased after UCMS (Fig. 4B, C). The rheobase of elicited single action potential in LH^Orx←mPFC^ neurons after UCMS was decreased (Fig. 4D, E). These finding suggested that the LH^Orx^-mPFC pathway was activated after UCMS.

To further verify how orexinergic neural projections from the LH to the mPFC responded to stress, we injected AAV2/9-hSyn-Orexin0.4 into the mPFC to examine the concentration of Orx under stress (Fig. 4F). The results showed that when mice received tail pinching and RS stimulation, Orx signaling in the mPFC immediately increased, and when the stimulation ended, Orx signaling decreased to normal levels (Fig. 4G, H). Therefore, our results show that stress can increase the activity of the LH^Orx^–mPFC pathway.

### The activation of the LH^Orx^–mPFC pathway induced anhedonia but not anxiety and despair behavior

As the LH^Orx^–mPFC pathway can be activated by stress, we examined whether the activation of this pathway is related to abnormal behavior caused by stress. We used designer receptors activated only by designer drugs (DREADD) to activate LH^Orx^ neurons projecting to the mPFC (Supplementary Fig. 3A). AAV2/Retro-hM3Dq-mCherry was injected into the mPFC, and AAV2/9-Hypocretin-CRE was injected into the LH to express hM3Dq in orexinergic neurons projecting to the mPFC (Supplementary Fig. 3B). The immunofluorescence of brain sections revealed the co-expression of hM3Dq and orexin-A in the LH (Supplementary Fig. 3C). CNO was injected intraperitoneally before behavioral testing to activate the LH^Orx^–mPFC neurons. The firing frequency of hM3Dq-expressing neurons in the LH significantly increased after CNO application (Supplementary Fig. 3D). In OFT, there was no significant difference in the time spent in the center zone and total distance between two group (Supplementary Fig. 4E, M). Activation of the LH^Orx^–mPFC pathway did not decrease the time spent in the open arms of the EPM (Supplementary Fig. 4F). Further, activating LH^Orx^ neurons projecting to the mPFC could not decrease the latency to feed in either the novelty or home cages (Supplementary Fig. 4G). Additionally, there was no significant difference in immobility time between mCherry-expressing mice and hM3Dq-expressing mice in either the FST or TST (Supplementary Fig. 4H, I). However, hM3Dq-expressing mice spent less time grooming during in the SST and decreased sucrose preference indicators, such as volume consumed, drinking duration and bouts (Supplementary Fig. 4J–L). These results suggested that activation of the LH^Orx^–mPFC pathway specificity induces anhedonia, but not anxiety or despair in naive mice.

**Fig. 5:**
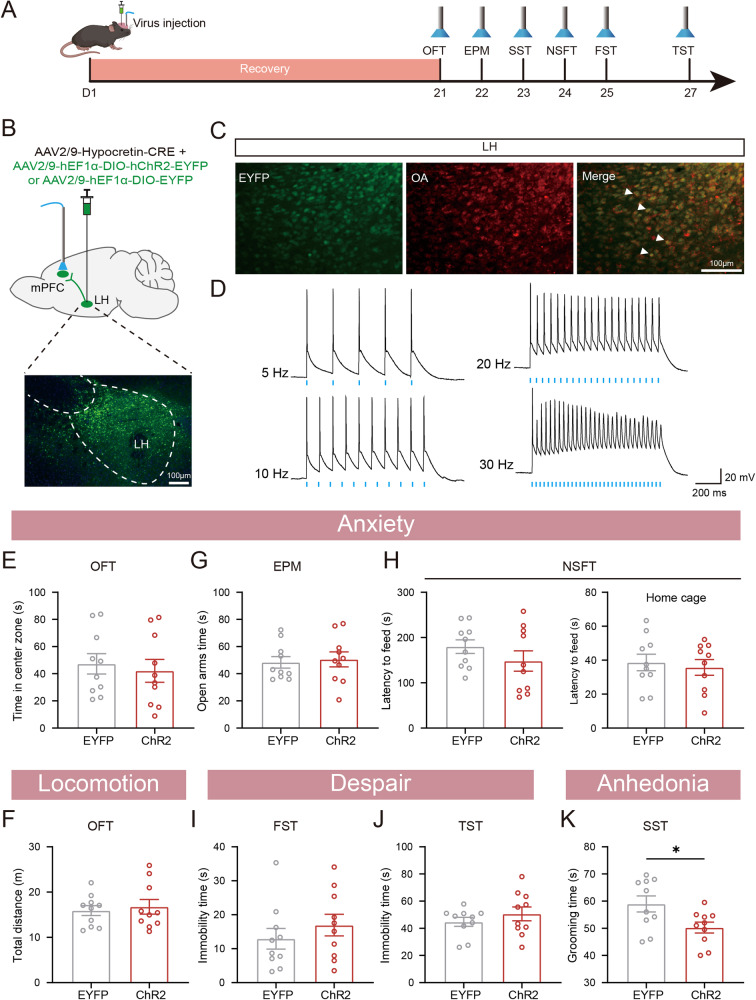
The optogenetic activation of the LH^Orx^–mPFC pathway induced anhedonia but not anxiety and despair behavior. **A** Experimental timeline of optogenetic activation of LH^Orx^-mPFC pathway (Blue light pluses at 465 nm, 30 Hz, 5 ms, 5 mW). **B** Top: Schematic of viral injection. Bottom: Representative expression of ChR2 virus. **C** Representative image of co-expression of EYFP with neurons expressing Orexin-A immunoreactivity in LH. **D** Representative recording of the action potential firing of Orx-expressing neurons in response to 5, 10, 20, and 30 Hz light photostimulation. Activating the LH^Orx^-mPFC pathway did not affect anxiety-like (**E**: time in center of the OFT, **G**: time in open arms of the EPM, **H**: latency to feed in the NSFT), despair-like phenotypes (**I**: immobility time in the FST, **J**: immobility time in the TST) and locomotion (**F**: total distance in the OFT). **K** Activating LH^Orx^-mPFC pathway decreased grooming time in the SST. **F**–**L** *n* = 10. Data are shown as mean ± S.E.M., unpaired *t* test, *P < 0.05.

Chemogenetic techniques using CNO allow for control at the scale of hours, while optogenetics enables precise temporal control to the millisecond level by manipulating light. Therefore, we utilized an optogenetic approach to selectively activate the terminals of LH^Orx^ neurons projecting to the mPFC (Fig. 5A). AAV2/9-Hypocretin-CRE and AAV2/9-DIO-ChR2-EYFP were injected into the LH to express ChR2 in the LH orexinergic neurons, and an optical fiber was implanted into the mPFC (Fig. 5B). Blue light was delivered into the mPFC to activate the orexinergic terminal in the LH during the behavioral test. The immunofluorescence of brain sections revealed the co-expression of EYFP and orexin-A in the LH (Fig. 5C). To verify the function of ChR2, electrophysiological methods was performed. The firing frequency of ChR2-expressing neurons in the LH could be obviously increased by blue light up to frequencies of 30 Hz (Fig. 5D). In addition, after stimulation with blue light, we found a higher number of c-Fos expression cells in the LH with ChR2 compared to EYFP expression cells, which confirmed the functionality of the optogenetic probe (Supplementary Fig. 4A, B). The behavioral results showed that activation of the LH^Orx^–mPFC pathway did not alter the time spent in the central zone of the OFT, and the total distance traveled did not significantly differ between the ChR2 and EYFP groups (Fig. 5E, F). Further, activating the ChR2-expressing terminals did not change the time spent in the open arms of the EPM (Fig. 5G). In addition, there was no difference in the latency to feed in either the novelty or home cages between the two groups (Fig. 5H). Furthermore, activating orexinergic projection terminals in the mPFC did not regulate immobility in either the FST or TST (Fig. 5I, J). However, the optogenetic stimulation of orexinergic projection terminals in the mPFC during behavioral tests decreased grooming time in the SST (Fig. 5K). These results suggest that activation of the LH^Orx^–mPFC pathway selectively induces anhedonia, while no significant effects on anxiety and despair behavior were observed.

### The chemogenetic inhibition of the LH^Orx^–mPFC pathway alleviated UCMS-induced anhedonia, but not anxiety and despair

**Fig. 6:**
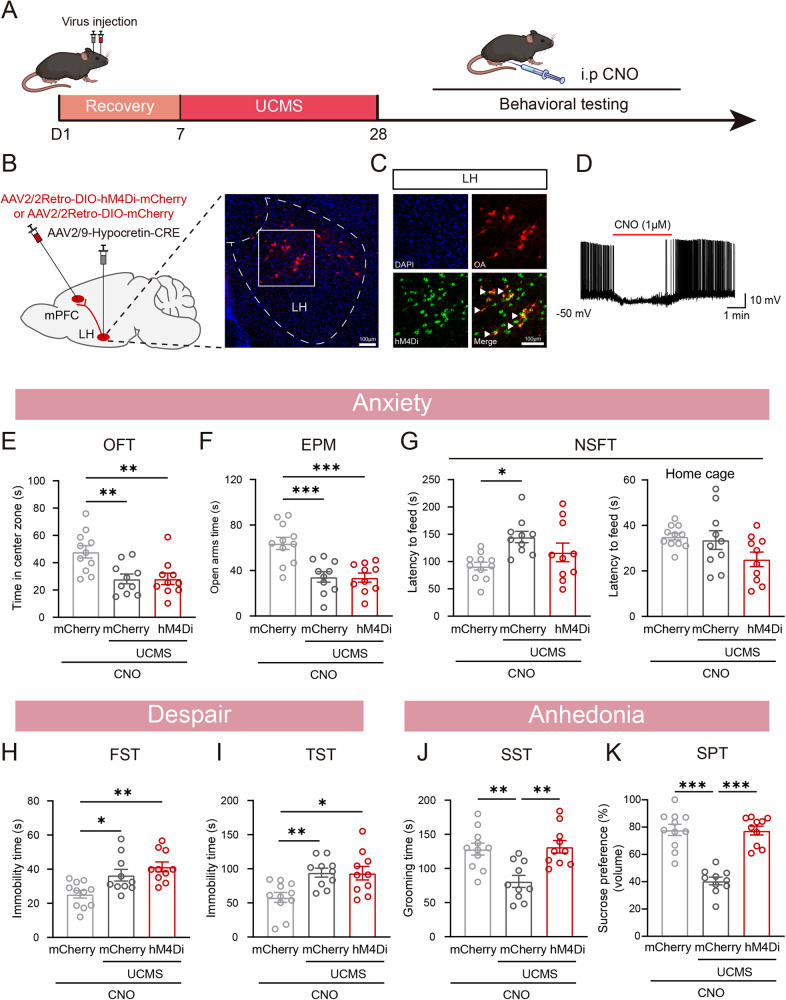
The chemogenetic inhibition of the LH^Orx^–mPFC pathway alleviated UCMS-induced anhedonia, but not anxiety and despair. **A** Experimental timeline of chemogenetic inhibition of LH^Orx^-mPFC pathway, CNO injections before behavioral tests (3.3 mg/kg). **B** Left: Schematic of viral injection. Right: Representative image of virus expression in the LH. **C** Representative image of co-expression of mCherry with neurons expressing Orexin-A immunoreactivity in LH. **D** The representative trace showed that the orexinergic neurons expressing hM4Di inhibited after CNO bath application. Inhibiting the LH^Orx^-mPFC pathway Time did not prevent anxiety-like (**E** time in center of the OFT, **F** time in open arms of the EPM, **G** latency to feed in the NSFT), despair-like phenotypes (**H** immobility time in the FST, **I** immobility time in the TST). **J** Inhibiting LH^Orx^-mPFC pathway increased the grooming time in the UCMS mice in SST. **K** In the SPT, inhibiting LH^Orx^-mPFC pathway increased volume consumed in the UCMS mice. *n*_(Ctrl + mCherry)_ = 11, *n*_(UCMS + mCherry)_ = 10, *n*_(UCMS + hM4Di)_ = 10. Data are shown as mean ± S.E.M., one-way ANOVA, *P < 0.05, **P < 0.01, ***P < 0.001.

Based on the fact that the LH^Orx^–mPFC pathway can induce anhedonia in mice, we examined whether UCMS-induced anhedonia was caused by the overactivation of the LH^Orx^–mPFC pathway, and whether silencing of the LH^Orx^–mPFC pathway was sufficient to alleviate UCMS-induced anhedonia (Fig. 6A). To verify this, we used DREADD to inhibit LH^Orx^ neurons projecting into the mPFC. AAV2/Retro-hM4Di-mCherry was injected into the mPFC, and AAV2/9-Hypocretin-CRE was injected into the LH to express hM4Di in orexinergic neurons projecting to the mPFC (Fig. 6B). The immunofluorescence of brain sections revealed the co-expression of mCherry and orexin-A in the LH (Fig. 6C). CNO was injected intraperitoneally before behavioral testing to inhibit the LH^Orx^–mPFC neurons. The firing frequency of hM4Di-expression neurons in the LH significantly decreased after CNO application (Fig. 6D). In the OFT, there was no significant difference in the time spent in the center zone between the mCherry-expressing mice and the hM4Di-expressing mice, and there was no effect on the total distance traveled in either of the two groups (Fig. 6E and Supplementary Fig. 5A). Inhibition of the LH^Orx^–mPFC neurons did not reduce the time spent in the open arms of the EPM (Fig. 6F). In the NSFT, the chemogenetic inhibition of this pathway did not regulate the latency time in either the novelty or home cage (Fig. 6G). Additionally, there was no significant difference in immobility time between mCherry-expressing mice and hM4Di-expressing mice in either the FST or TST (Fig. 6H, I). Compared to the mCherry-expressed mice, the hM4Di-expressing mice spent significantly more time grooming during the SST and increased volume consumed in the SPT (Fig. 6J, K), but not alleviated drinking duration and bouts in the SPT (Supplementary Fig. 5B). Therefore, we conclude that the LH^Orx^–mPFC pathway plays a crucial role in mediating UCMS-induced anhedonia, but has no involvement in anxiety or despair behaviors.

Since we found the orexinergic neurons in the LH could project to the ACC, part of the prefrontal cortex, we discovered that the number of c-Fos-positive cells in the ACC increased after RS, indicating that the ACC may be involved in the regulation of stress (Supplementary Fig. 6A–C). To verify whether the LH^Orx^–ACC pathway can induce anhedonia in mice, DREADD was used to inhibit the LH^Orx^ neurons projecting to the ACC (Supplementary Fig. 6D–F). In the SPT, there was no significant difference in sucrose preference indicators, such as volume consumed, drinking duration and bouts between mCherry-expressing mice and hM4Di-expressing mice after UCMS (Supplementary Fig. 6G–I), indicating that the LH^Orx^–ACC pathway did not involve in the regulation of anhedonia.

### Blocking OX1R or OX2R in the mPFC alleviated UCMS-induced anhedonia, but not anxiety and despair

**Fig. 7:**
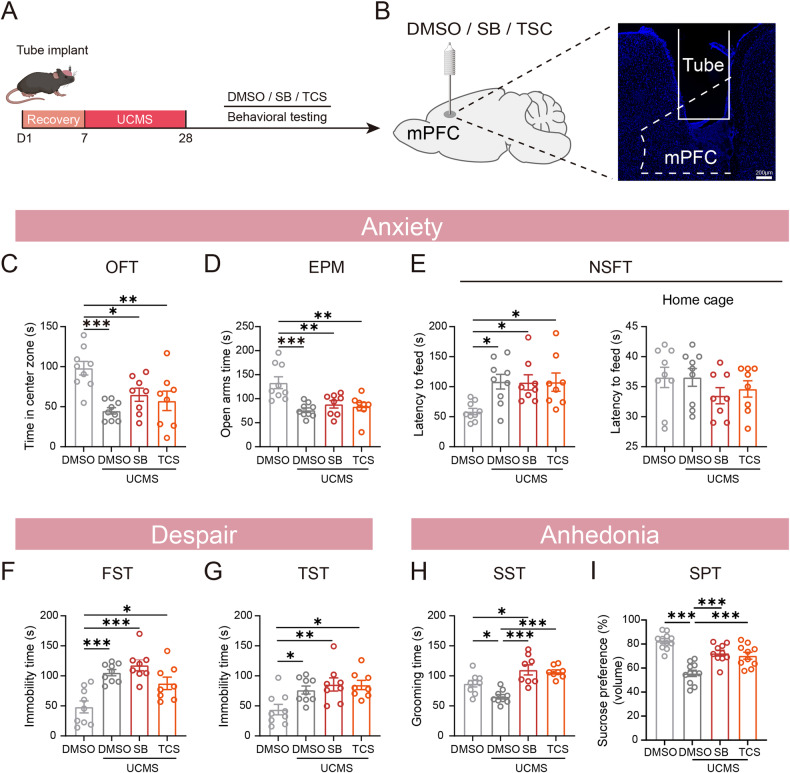
Blocking OX1R or OX2R in the mPFC alleviated UCMS-induced anhedonia, but not anxiety and despair. **A** Experimental timeline of microinjection blocking the LH^Orx^-mPFC pathway. **B** Left: Schematic of microinjection. Right: Representative image of tube in the mPFC. Blocking either OX1R or OX2R did not prevent anxiety-like (**C** time in center of the OFT, **D** time in open arms of the EPM, **E** Latency to feed in the NSFT), despair-like phenotypes (**F** immobility time in the FST, **G** Immobility time in the TST). **H** In the UCMS mice, blocking OX1R or OX2R increased the grooming time in the SST. **I** Blocking OX1R or OX2R increased volume consumed in the SPT after UCMS. *n*_(Ctrl + DMSO)_ = 11, *n*_(UCMS + DMSO)_ = 11, *n*_(UCMS + SB)_ = 10, *n*_(UCMS + TCS)_ = 10. Data are shown as mean ± S.E.M., one-way ANOVA, *P < 0.05, **P < 0.01, ***P < 0.001.

We confirmed that inhibition of the LH^Orx^–mPFC pathway alleviated UCMS-induced anhedonia. To explore the subtypes of Orx receptors in the mPFC involved in modulation of anhedonia, we microinjected Ox1R antagonist SB, Ox2R antagonist TCS and DMSO separately into the mPFC 30 min before behavioral tests (Fig. 7A, B). In the OFT, compared to UCMS-DMSO group, there was no difference in the time spent in the central zone between the UCMS-SB group and the UCMS-TCS group, and there was no effect on the total distance traveled in either of the four groups (Fig. 7C, Supplementary Fig. 7A). Blocking the Ox1R and Ox2R in the mPFC did not increase the time spent in the open arms of the EPM in UCMS mice (Fig. 7D). In the NSFT, blocking the Ox1R and Ox2R in the mPFC did not reduce the latency time in either the novelty or home cage in UCMS mice (Fig. 7E). Additionally, compared to UCMS-DMSO, there was no significant difference in immobility time in both UCMS-SB and UCMS-TCS in either the FST or TST (Fig. 7F, G). Compared to the UCMS-DMSO mice, the UCMS-SB mice and UCMS-TCS mice spent significantly more time grooming during the SST and increased volume consumed and drinking bouts in the SPT (Fig. 7H, I and Supplementary Fig 7B Right), but not alleviated drinking duration in the SPT (Supplementary Fig. 7B Left). Accordingly, we concluded that the orexinergic terminals alleviated UCMS-induced anhedonia through Ox1R and Ox2R in the mPFC.

## Discussion

In this study, we confirmed that UCMS induced anxiety, despair, and anhedonia in mice. Simultaneously, c-Fos immunopositive cells were increased in the LH after mice were exposed to stress, indicating that stress could activate the LH. The in vivo fiber photometry results indicated that orexinergic neurons were active after UCMS-induced anxiodepression. The mPFC could also be activated after stress, as it is downstream of the LH^Orx^ neurons. We verified that the neurons in the LH projecting to the mPFC were mainly orexinergic neurons and that the concentration of Orx in the mPFC increased significantly when the animals were exposed to stress, indicating that orexinergic neuron activity increased during stress. We demonstrated that the LH^Orx^-mPFC pathway was activated after UCMS. Activation of the LH^Orx^–mPFC pathway induced anhedonia, whereas both inhibition of the LH^Orx^–mPFC pathway and blocking the Ox1R and Ox2R in the mPFC could alleviate UCMS-induced anhedonia. However, both the activation and the chemogenetic inhibition of the LH^Orx^–mPFC pathway, as well as blocking the Ox1R and Ox2R in the mPFC, did not yield a significant effect on anxiety or despair behaviors. Therefore, UCMS-induced activation of the LH^Orx^–mPFC pathway specifically drove anhedonia through Ox1R and Ox2R, but not anxiety or despair.

It is now well established that Orx and its receptors are involved in the pathophysiology of depression, but their roles are still controversial. Consistent with some previous studies, we found that LH orexinergic neurons can be activated by UCMS and chronic social defeat stress (CSDS) [28]. Overactivation of the Orx system may be a significant contributing factor to anxiodepression. Recent studies have shown that the inhibition of the Orx system by the intraperitoneal injection of an OX1R antagonist produces an antidepressant-like effect in healthy mice [29]. In addition, daily treatment with a dual Orx receptor antagonist counteracts UCMS- and CSDS-induced depression-like behaviors and enhances social interactions [30, 31]. Therefore, Orx receptor antagonists have been investigated as potential targets for the treatment of major depressive disorder (MDD) [32], with a phase 2b study suggesting that an OX2R antagonist had an antidepressant effect on patients with MDD [33]. Thus, the Orx system could be evaluated as an option for the clinical treatment of depression in humans. Building on this foundation, the present study further explored the neural circuits through which the Orx system regulates anxiety and depression.

LH orexinergic neurons are extensively interconnected with multiple brain regions and play a central role in a wide range of behaviors. The mPFC is associated with stress-induced cognitive and emotional impairments and accepts many projections from LH orexinergic neurons [34, 35]. Here, we found that the orexinergic neurons predominantly project to the glutamatergic neurons in the mPFC. The excitement of the mPFC^Glu^ neurons was increased after UCMS, while UCMS stressors increased Orx release in the mPFC. LH orexinergic neurons target the layer V pyramidal neurons in the mPFC and induce excitatory postsynaptic currents [10]. Furthermore, activation of the mPFC can inhibit natural reward-related behavior [36]. Although previous studies found that increased excitability in the mPFC was associated with anxiety, depression, and anhedonia [37], we independently modulated the LH^Orx^–mPFC pathway and found that activating this pathway could only induce anhedonia, while inhibiting this pathway could alleviate UCMS-induced anhedonia. This was consistent with fMRI studies showing increased mPFC reactivity in adolescents with anhedonia who experienced higher negative effects in their daily lives [38]. However, modulation of the activity of LH^Orx^–mPFC pathway did not affect anxiety or despair. This may be due to the fact that the neurons in the mPFC can be divided into multiple subtypes according to their projection location or cell type, and they can regulate different behaviors. For example, LHb activation inhibits the mPFC, which controls social competitiveness and reinforces retreating behavior in contests, leading to a depression-like state associated with a loss of social status [27]. Meanwhile, the optical activation of the BLA–mPFC pathway reverses CSDS-induced social avoidance [39]. Therefore, projections from LH orexinergic neurons to the mPFC may only regulate anhedonia.

Anhedonia is characterized by a persistent and pronounced reduction in interest or pleasure in nearly all daily activities that occur regularly. Diagnosing and treating anhedonia can be challenging because of its complex nature. Presently, the treatment of depression strongly emphasizes decreasing negative emotions rather than elevating the deficiency of positive emotion [40]. Increased reward pursuit does not induce an anticipatory pleasure experience in social anhedonia [41]. Current pharmacological or psychological therapies do not address motivation or reward processing deficits in anhedonia [42]. Owing to its noteworthy clinical influence on the prognosis of depression, the development of a targeted therapy for anhedonia is crucial. In addition to depression, anhedonia is also a transdiagnostic symptom of other psychiatric disorders, including posttraumatic stress disorder, eating disorders, and substance use disorder [42–45]. In our study, we used the SST and SPT to measure anhedonia behavior. A decrease in grooming time in the SST is related to anhedonia [19, 20, 46, 47]. Recent studies have focused on validating the neural circuitry underlying anhedonia. During chronic mild stress, the optogenetic activation of the VTA dopaminergic (DA) neurons increased sucrose preference in the ChR2- and EYFP-treated mice [48]. The optogenetic inhibition of the arcuate nucleus of the hypothalamus (ARH) pro-opiomelanocortin (POMC) neurons projecting to the VTA^DA^ circuit alleviated anhedonia in chronic restraint stress (CRS) [49]. Our findings indicate that the LH^Orx^–mPFC pathway is a novel and specific target for the treatment of anhedonia.

While we collectively validated the significant role of the LH^Orx^–mPFC neurocircuit in stress-induced anhedonia using various approaches, including optogenetics, fiber photometry recordings, and behavioral studies, there were some limitations to our study. The mice were tested using multiple behavioral assays within a relatively short period and in the same consistent order. However, to minimize the possible carryover effects of the different behavioral tests, the sequence of the tests was arranged from the least to most stressful [13, 25]. Therefore, further investigation is warranted to elucidate the specific roles of mPFC neurons in the amelioration of anhedonia.

Our study revealed that stress leads to increased activity in the LH and mPFC. Furthermore, we observed that LH neurons projecting to the mPFC received predominant inputs from orexinergic neurons, and demonstrated that orexinergic neurons in the LH make functional synaptic connections in the mPFC. Moreover, the concentration of Orx in the mPFC increased in mice exposed to stress and the effect of LH^Orx^–mPFC pathway was activated after UCMS. To further support the functional relevance of the LH^Orx^–mPFC pathway, optogenetic and chemogenetic methods were used to demonstrate that activation of the LH^Orx^–mPFC pathway can specifically reduce anhedonia in depressed mice. Furthermore, the inhibition of LH^Orx^–mPFC projections specifically ameliorated UCMS-induced anhedonia. Additionally, blocking the Ox1R and Ox2R in the mPFC alleviated UCMS-induced anhedonia. Our results clarified that the overactivation of the LH^Orx^–mPFC pathway mediated chronic stress-induced anhedonia through Ox1R and Ox2R, but had no significant effect on anxiety or despair behaviors.

### Supplementary information

Supplemental Information

## Data Availability

The data presented in this study are available upon request from the corresponding authors.
