# Supplementary material for: Lateral hypothalamus orexinergic projection to the medial prefrontal cortex modulates chronic stress-induced anhedonia but not anxiety and despair
Source: Transl Psychiatry. 2024 Mar 16;14:149. doi: 10.1038/s41398-024-02860-9 (PMC10944479; doi:10.1038/s41398-024-02860-9)
Supplement: Supplementary file 1 — Supplemental Information [file 41398_2024_2860_MOESM1_ESM.docx]

**Supplementary Information**

**
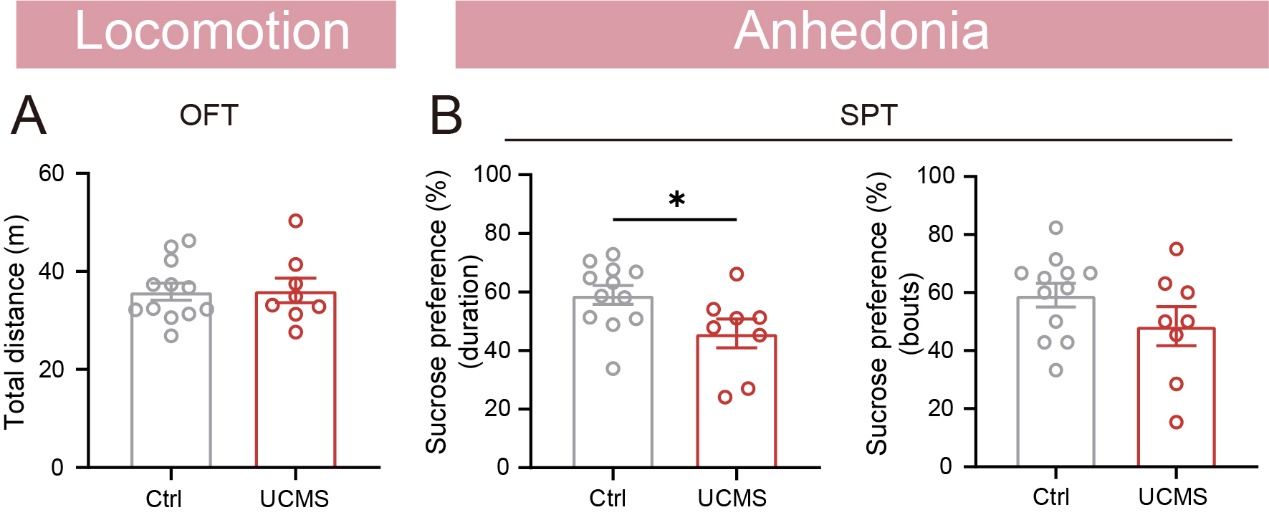
**

Supplementary Fig. 1. UCMS drove anhedonia, but not locomotion.

A. UCMS had no effects on the total distance traveled in the OFT. B. Left: UCMS decreased drinking duration in the SPT. Right: UCMS did not affect drinking bouts in the SPT. *n*_(Ctrl)_ = 12, *n*_(UCMS)_ = 8. Data are shown as mean ± S.E.M., unpaired Student’s t*-*test, **P* < 0.05.


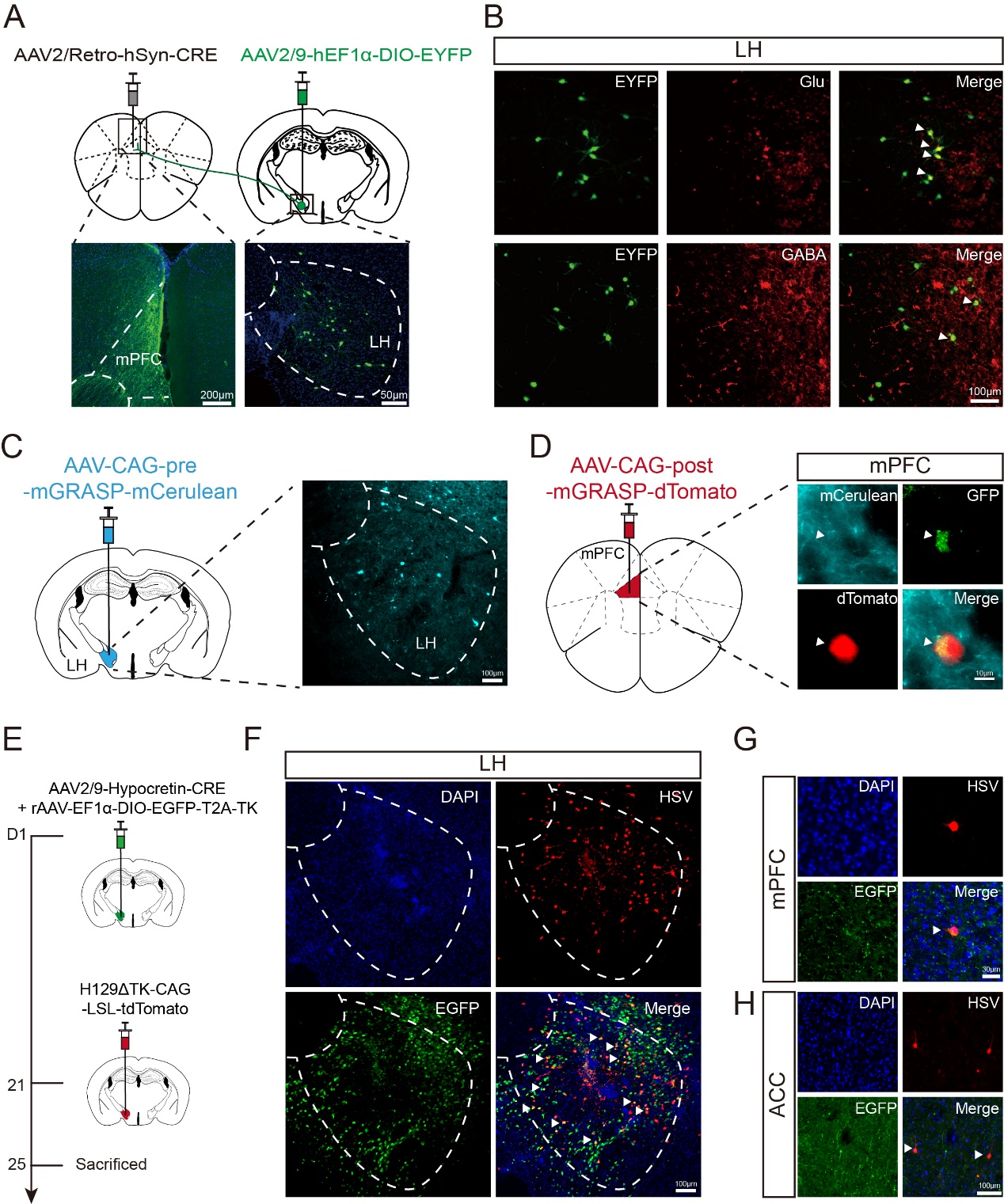


Supplementary Fig. 2. The connection between the LH and the mPFC.

A: Top: schematic viral injection plan; Bottom: representative photomicrographs of the mPFC and the LH injection site. The dashed white lines indicated boundaries of the mPFC and LH. B. Representative photomicrographs of EYFP expression overlapped with neurons expressing Glu (top) and GABA (bottom). C. Left: Schematic of viral injection in LH. Right: Representative photomicrographs of virus in the LH. D. Left: Schematic of viral injection in mPFC. Right: Representative photomicrographs of mGRASP system. E. The experiment protocols for HSV injection and schematic viral injection plan. F. Representative photomicrographs of HSV in the LH. G. Representative photomicrographs of HSV in the mPFC. H. Representative photomicrographs of HSV in the ACC.


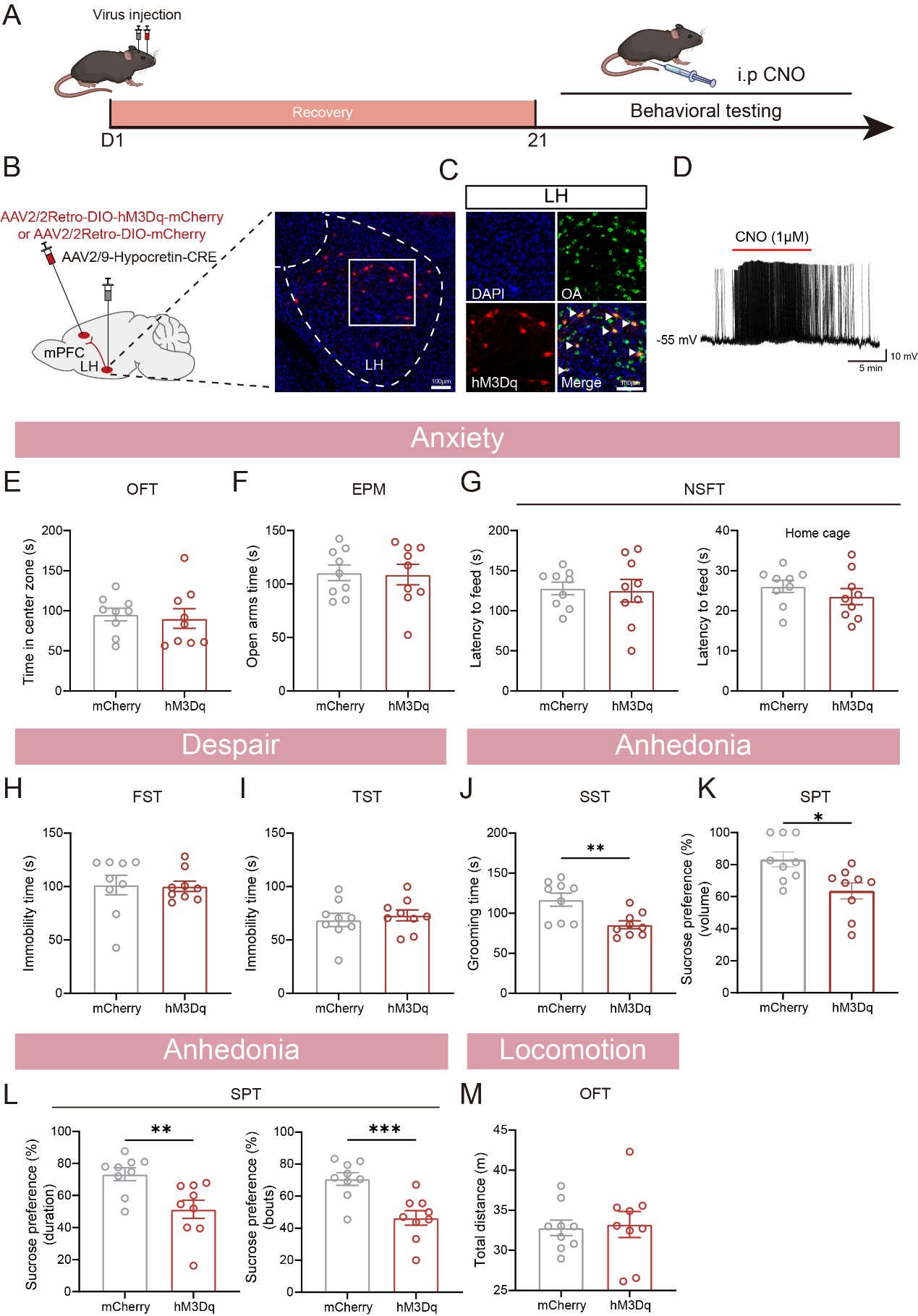


Supplementary Fig. 3. The chemogenetic activation of the LH^Orx^–mPFC pathway induced anhedonia but not anxiety and despair behavior.

A. Experimental timeline of chemogenetic activation of LH^Orx^-mPFC pathway. CNO injections before behavioral tests (3.3mg/kg). B. Left: Schematic viral injection plan. Right: Representative expression of hM3Dq virus. C: Representative image of co‑expression of hM3Dq with neurons expressing Orexin-A immunoreactivity in LH. D. The representative trace showed that the orexinergic neurons expressing hM3Dq were activated after CNO bath application. E-I. Activating the LH^Orx^-mPFC pathway did not affect anxiety-like (E: time in center of the OFT, F: time in open arms of the EPM, G: latency to feed in the NSFT), despair-like phenotypes (H: immobility time in the FST, I: immobility time in the TST). J-L. Activating LH^Orx^-mPFC pathway decreased grooming time in the SST (K) and source preference indicators, such as volume consumed, drinking duration and bouts in the SPT (J). M. Activating the LH^Orx^-mPFC pathway did not affect the total distance traveled in the OFT. *n* = 9. Data are shown as mean ± S.E.M., unpaired t test, **P* < 0.05, ***P* < 0.01, ****P* < 0.001.


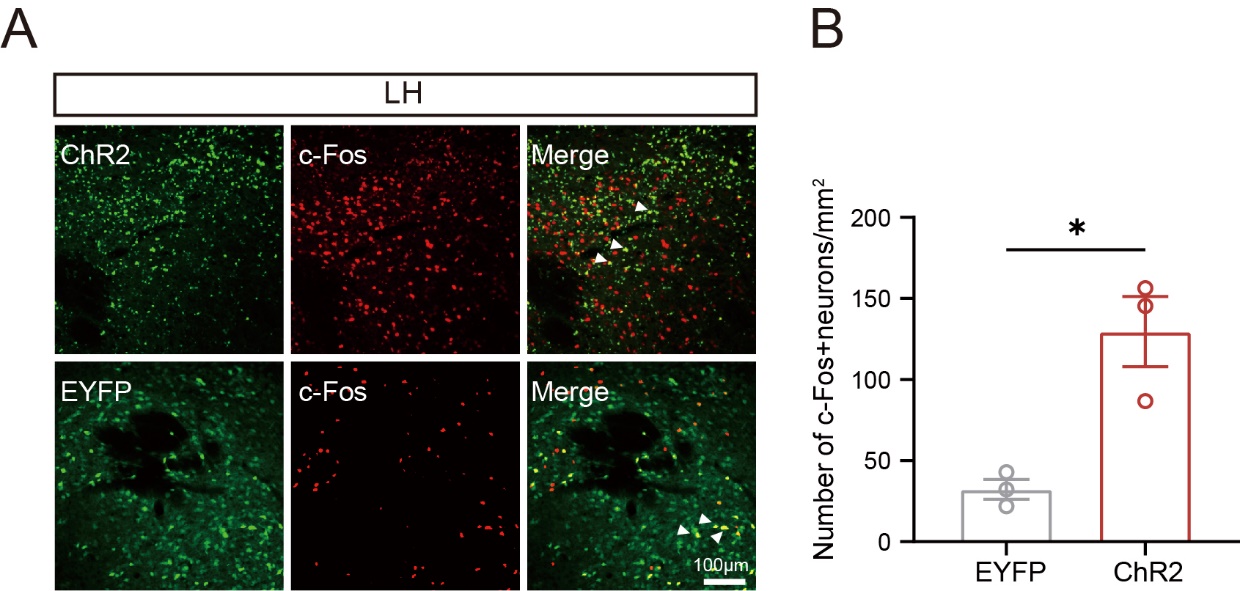


Supplementary Fig. 4. Blue light exposure enhanced the activity of ChR2-expressing neurons.

A. Representative overlap of ChR2 or EYFP virus with neurons expressing c-Fos after blue light was delivered into the LH. B. The density of c-Fos^+^ neurons (n = 3, *t* = 4.33, df = 4, 32.37 ± 6.059 vs 129.6 ±21.61, *P*=0.0123, unpaired Student’s t*-*test; Data are shown as mean ± S.E.M., unpaired t test, **P* < 0.05).


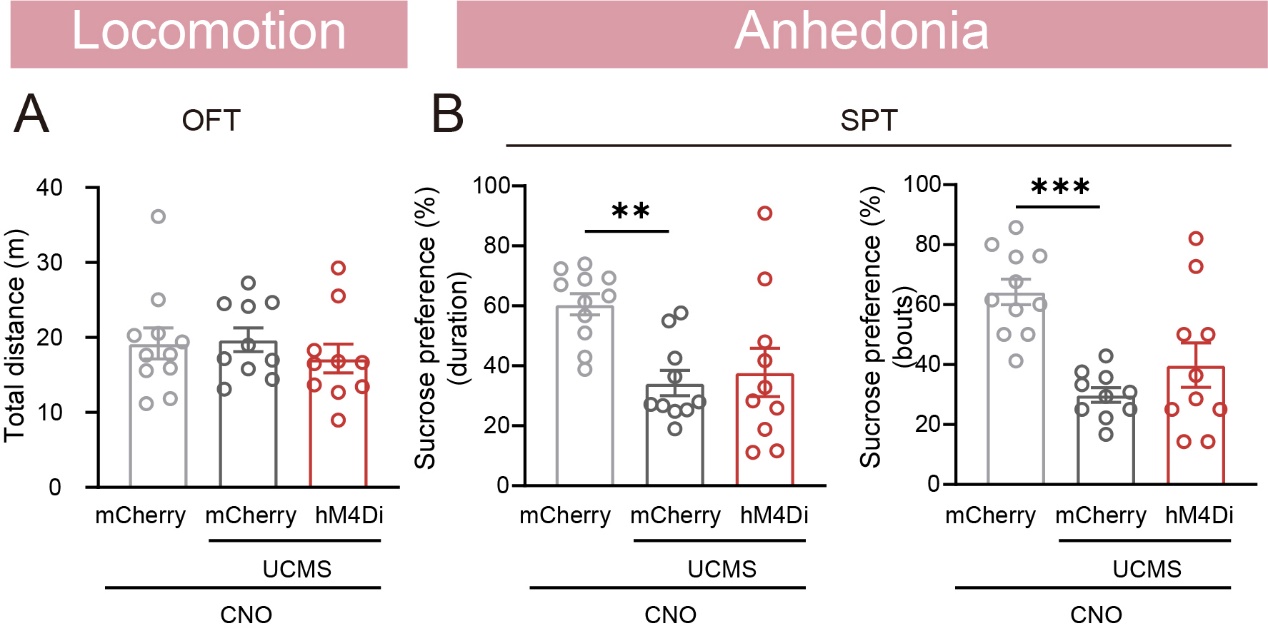


Supplementary Fig. 5. Chemogenetic inhibition of LH^Orx^-mPFC pathway did not affect drinking duration and bouts in the SPT and locomotion in the OFT.

A. Inhibiting the LH^Orx^-mPFC pathway did not affect the total distance traveled in the OFT. B. Inhibiting the LH^Orx^-mPFC pathway did not change the drinking duration and bouts in the SPT. *n* _(Ctrl + mCherry)_ = 11, *n* _(UCMS + mCherry)_ = 10, *n* _(UCMS + hM4Di)_ = 10. Data are shown as mean ± S.E.M., one-way ANOVA, ***P* < 0.01, ****P* < 0.001.


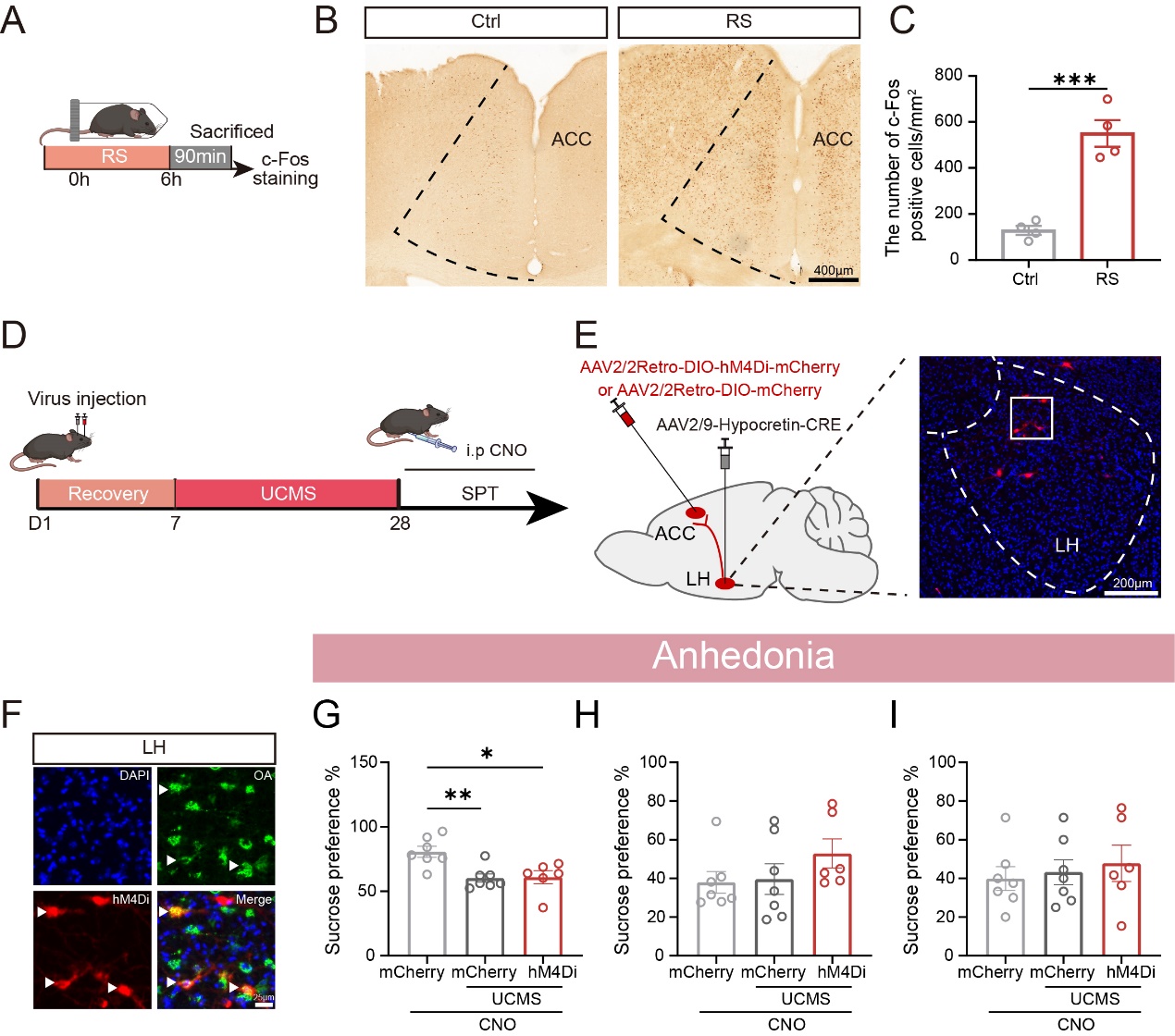


Supplementary Fig. 6. LH^Orx^-ACC pathway could not prevent UCMS-induced anhedonia.

A. The experimental protocol for the activity of the ACC after RS. B. Representative c-Fos positive cells in the LH of Ctrl and RS mice. C. The density of c-Fos positive cells in the ACC region (n = 4). D. Experimental timeline of chemogenetic inhibition of LH^Orx^-ACC pathway, CNO injections before behavioral tests (3.3mg/kg). E. left: Schematic viral injection plan. right: Representative image of virus expression in the LH. F. Representative image of co‑expression of mCherry with neurons expressing Orexin-A immunoreactivity in LH. G-I. Inhibiting the LH^Orx^-mPFC pathway did not change the volume consumed (G), drinking duration (H) and bouts (I) in the SPT. *n* _(Ctrl + mCherry)_ = 7, *n* _(UCMS + mCherry)_ = 7, *n* _(UCMS + hM4Di)_ = 6. Data are shown as mean ± S.E.M., one-way ANOVA, **P* < 0.05, ***P* < 0.01, ****P* < 0.001.


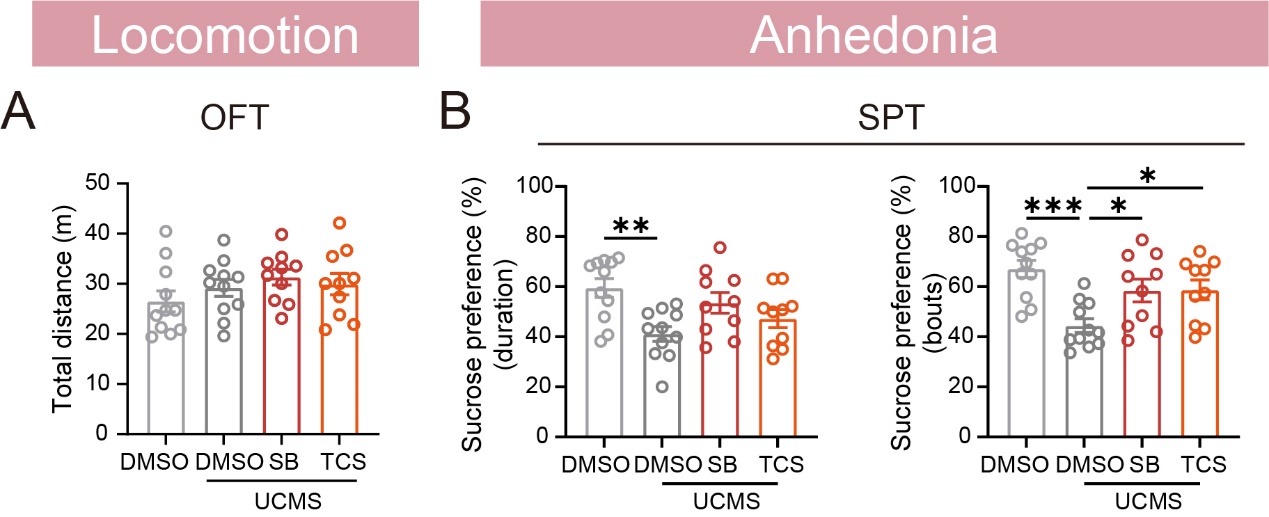


Supplementary Fig. 7. Blocking OX1R or OX2R in the mPFC alleviated drinking bouts in the SPT, but did not affected locomotion in the OFT.

A. Blocking OX1R or OX2R had no effects on the total distance traveled in the OFT. B. Left: Blocking OX1R or OX2R did not affected drinking duration in the SPT. Right: Blocking OX1R or OX2R increased drinking bouts in the SPT after UCMS.
